# Association between radiological CDSS availability and quality of imaging referrals graded using RI-RADS

**DOI:** 10.1016/j.isci.2026.117601

**Published:** 2026-09-25

**Authors:** Yi Xiang Tay, Wenlu Hou, Shane J. Foley, Ronan Killeen, Marcus E.H. Ong, Jeremy C.P. Wee, Ivan S.Y. Chua, Bingyi Wang, Peter Doran, Robert Chun Chen, Lai Peng Chan, Jonathan P. McNulty

**Affiliations:** 1 Radiography and Diagnostic Imaging, School of Medicine, University College Dublin, Belfield, Dublin, Ireland; 2 Radiography Department, Allied Health Division, Singapore General Hospital, Outram Road, Singapore, Singapore; 3 Department of Neuroradiology, Division of Radiological Sciences, Singapore General Hospital, Outram Road, Singapore, Singapore; 4 National Neuroscience Institute, 1 Hospital Boulevard, Singapore, Singapore; 5 St Vincent’s University Hospital, Elm Park, Dublin, Ireland; 6 School of Medicine, University College Dublin, Belfield, Dublin, Ireland; 7 Department of Emergency Medicine, Division of Medicine, Singapore General Hospital, Outram Road, Singapore, Singapore; 8 Health Services Research and Population Health, Duke-NUS Graduate Medical School, 8 College Road, Singapore, Singapore; 9 Clinical Research Centre, School of Medicine, University College Dublin, Belfield, Dublin, Ireland; 10 Department of Musculoskeletal Radiology, Division of Radiological Sciences, Singapore General Hospital, Outram Road, Singapore, Singapore

**Keywords:** clinical decision support system, radiology, implementation science, emergency department, diagnostic imaging

## Abstract

Deficiencies in clinical information within imaging referrals can affect imaging justification and the quality of radiology reporting. This pragmatic controlled before-and-after implementation study evaluated cervical and lumbar spine X-ray referral quality in an emergency department before and after the availability of a standalone clinical decision support system (CDSS) alongside existing workflows. Referrals were assessed using the reason for exam imaging reporting and data system (RI-RADS), grades A–D. Approximately three-quarters were classified as deficient, grade D. Logistic regression and mixed-effects modeling demonstrated no statistical significance between-group effect, although temporal confounding prevents firm conclusions regarding CDSS effectiveness. Both groups showed reductions in deficient referrals over time, with no additional improvement associated with CDSS availability beyond observed secular trends. These findings highlight challenges associated with standalone CDSS implementation and emphasize the importance of workflow and health-technology integration when designing decision support to improve imaging referral quality.

## Highlights

• 3/4 of the cervical and lumbar spine X-rays were classified as deficient (grade D)
• Availability of CDSS did not improve RI-RADS beyond secular improvement
• Apparent limitations in implementing standalone CDSS alongside existing practices
• Use of structured indications and CDSS design weaknesses warrants further attention

## Graphical abstract

**Figure:**
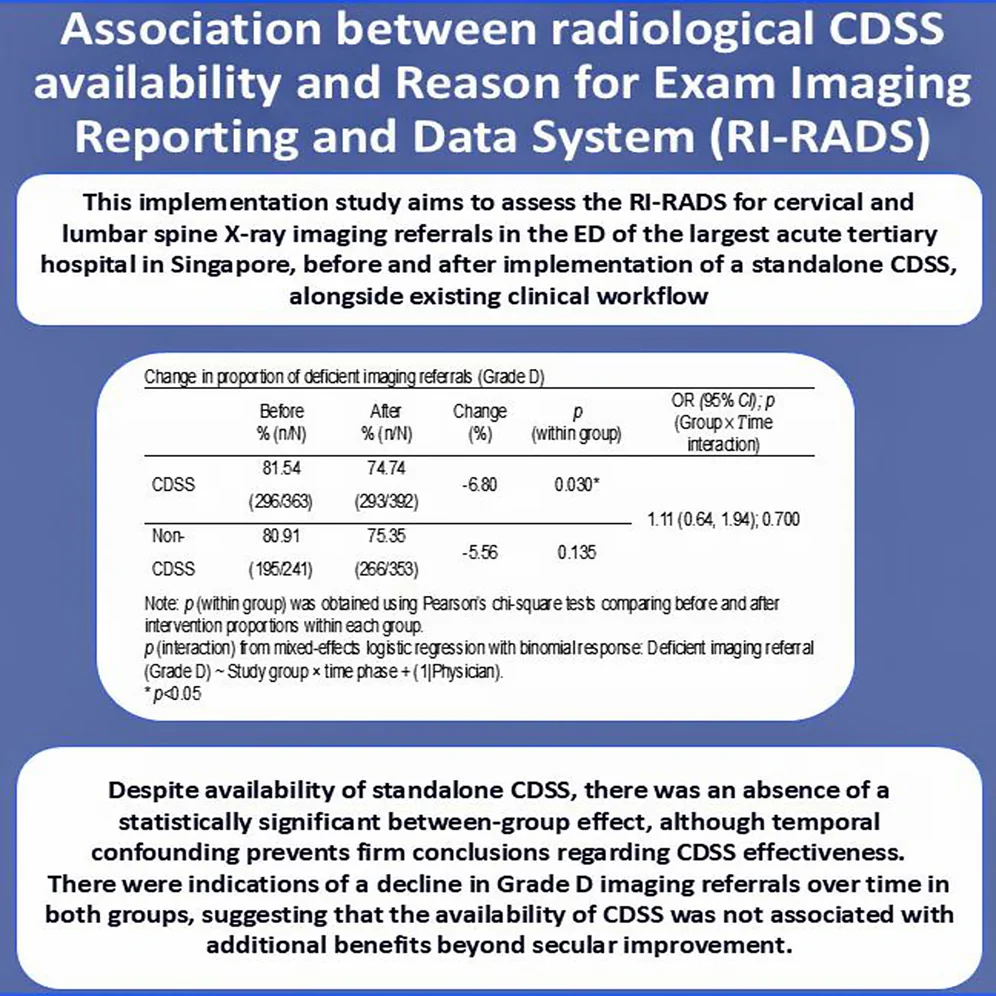

## Teaser

Health sciences; Medicine; Medical specialty; Radiology; Health informatics; Diagnostics

## Introduction

Radiology positively impacts patient outcomes by enhancing clinical workup through the refinement of differential diagnoses derived from history-taking, physical examinations, and laboratory test results.^1^ To many patients, radiology or diagnostic imaging is a pathway for an answer to a clinical question. Indeed, this is where radiology delivers value to healthcare recipients (patients) and their “immediate customers” (referring clinicians) by providing prevention, detection, diagnosis, prognosis, and therapy monitoring.^1^ Notably, the provision and dissemination of information in a radiological report reflects a form of value delivery. Consequently, radiologists, or radiographers in some jurisdictions, must provide accurate diagnostic interpretations in an accessible format, accompanied by pertinent recommendations for further imaging, while ensuring that information (addressing clinical needs) desired by the referring clinician are embedded within the report.^2^^,^^3^

However, many involved in reporting allude to the fact that they are often forced to perform reporting in a state of relative information starvation.^4^ This includes deficiencies in the amount of clinical information and key elements of important clinical data accompanying the imaging referral.^4^ As such, the reporting radiologist or radiographer will have to spend excess time retrieving the patient files or contacting the referring clinicians, which in tandem increases their workload. While there may be many “justifications” for such omissions, they may negatively affect the specificity and usefulness of radiology reports, which is concerning given the rise in imaging referrals.^4^

A systematic review suggested that good clinical information positively impacts radiology reports and highlighted the need for creating criteria standards to guide clinicians’ requesting practices.^5^ The radiology report is a critical product generated to guide patient care; however, the value of the report depends on comprehensive and sufficient clinical information from referring clinicians, as it enhances both the pre-test probability and the post-test validity of imaging studies.^6^ Anecdotally, the quality of information provided for the imaging referrals still requires further action.

The reason for exam imaging reporting and data system (RI-RADS) was first proposed in 2019 to address the need for appropriate clinical information for imaging referrals.^7^ According to RI-RADS, pertinent clinical information should include clinical findings, impressions, and a diagnostic question.^7^ Through such a system, the provision of clinical information is standardized, to facilitate better recognition of the precise intent of the imaging referral.^7^ Furthermore, a Delphi study indicated that RI-RADS can be used as a standard quality assessment of imaging referrals and may improve the quality of patient care.^8^

Clinical decision support systems (CDSSs) have been advocated and recognized as tools to guide appropriate imaging when optimized and implemented appropriately.^9^ While prior research suggests a positive impact of CDSS on imaging appropriateness rates and associated outcomes, the effect of CDSS availability on the quality of imaging referral remains under-studied.^10^ Current clinician-facing CDSS including radiological systems necessitates the patient’s presence to gather and input essential clinical data for informed decision-making.^11^ Therefore, the quality of information in an imaging requisition is critical to generate accurate recommendations.

This study aimed to assess RI-RADS for emergency department (ED) X-ray imaging referrals in the largest acute tertiary hospital in Singapore, before and after implementation of a standalone CDSS, alongside existing clinical workflow. We focus on cervical and lumbar spine X-rays which were common examinations performed in the local ED and associated with low-value imaging.

## Results

### Physician characteristics

According to the designated roster, a total of 17 non-specialist ED physicians (seven medical officers and 10 locum medical officers) participated in the control group. In contrast, 23 non-specialist ED physicians (18 medical officers and five emergency medicine residents) were involved in the intervention group. Although actual CDSS utilization was not tracked at the physician level, a supplementary survey was conducted at the end of the intervention period. Of the 23 participating physicians surveyed, 11 (47.8%) reported using the CDSS.

### Control group: Imaging referral characteristics and RI-RADS grading

For the control group, the imaging referrals in the “before” phase consisted of 70 cervical spine X-ray and 171 lumbar spine X-rays while the “after” phase comprised 110 cervical spine X-ray and 243 lumbar spine X-rays (Table 1). The mean age of patients referred for cervical and lumbar spine X-rays was 61.7 ± 17.4 (before) and 58.4 ± 18.8 (after). Female patients were more predominant in both phases (before: female – 156 and male – 85 vs. after: female – 201 and male – 152). Most of the patients presented for non-traumatic clinical indications (before: non-trauma – 186 and trauma – 55 vs. after: non-trauma – 282 and trauma – 71).

**Table 1:** Quality of imaging referrals according to RI-RADS (control group)

	RI-RADS Grade	*n*/*N* (%)	Presence of clinical findings	Presence of impression	Presence of diagnostic question	Positive	Negative
**Before**							

Cervical spine X-ray	C	10/70 (14.3%)	10/10 (100%)	10/10 (100%)	0/10 (0%)	0/10 (0%)	10/10 (100%)
	D	60/70 (85.7%)	51/60 (85.0%)	8/60 (13.3%)	1/60 (1.7%)	1/60 (1.7%)	59/60 (98.3%)
Lumbar spine X-ray	B	1/171 (0.6%)	1/1 (100%)	1/1 (100%)	1/1 (100%)	1/1 (100%)	0/1 (0%)
	C	35/171 (20.5%)	33/35 (94.3%)	22/35 (62.9%)	14/35 (40%)	2/35 (5.7%)	33/35 (94.3%)
	D	135/171 (78.9%)	122/135 (90.4%)	9/135 (6.7%)	0/135 (0%)	18/135 (13.3%)	117/135 (86.7%)

**After**							

Cervical spine X-ray	B	1/110 (0.9%)	1/1 (100%)	1/1 (100%)	1/1 (100%)	0/1 (0%)	1/1 (100%)
	C	24/110 (21.8%)	23/24 (95.8%)	14/24 (58.3%)	10/24 (41.7%)	0/24 (0%)	24/24 (100%)
	D	85/110 (77.3%)	79/85 (92.9%)	6/85 (7.1%)	0/85 (0%)	0/85 (0%)	85/85 (100%)
Lumbar spine X-ray	C	62/243 (25.5%)	60/62 (96.8%)	33/62 (53.2%)	29/62 (46.8%)	9/62 (14.5%)	53/62 (85.5%)
	D	181/243 (74.5%)	176/181 (97.2%)	7/181 (3.9%)	1/181 (0.6%)	18/181 (9.9%)	163/181 (90.1%)

In the before phase (control), none of the imaging referrals were classified as *adequate* (grade A), with only one lumbar spine X-ray imaging referral classified as *barely adequate* (grade B). Most of the imaging referrals for both body regions (>75%) were deemed *deficient* (grade D), with the remaining (14.3% and 20.5%) classified as *considerably limited* (grade C). Only one of the cervical spine X-rays graded D had a positive radiological finding. These positive findings were observed across all lumbar spine X-rays, with the majority occurring in the examinations graded D (*n* = 18/135, 13.3%).

In the after phase, none of the imaging referrals received a grade A assessment, with only one cervical spine imaging referral rated as grade B. This trend mirrored that of the previous phase, where the majority (77.3% and 74.5%) of clinical information provided in the imaging referrals was deemed *deficient* (grade D), with the most frequently omitted information being the diagnostic question. Unlike the “before” phase, no positive finding was observed in the cervical spine X-rays. Majority of the positive findings for lumbar spine X-rays were reported in examinations graded D (*n* = 18/181, 9.9%), although the rates were higher for examinations graded C (*n* = 9/62, 14.5%).

### Intervention group: Imaging referral characteristics and RI-RADS grading

For the intervention group, the imaging referrals in the “before” phase consisted of 98 cervical spine X-ray and 265 lumbar spine X-rays while the “after” phase comprised 110 cervical spine X-ray and 282 lumbar spine X-rays (Table 2). The mean age of patients referred for both imaging was 58.6 ± 19.6 (before) and 58.8 ± 19.7 (after). Female patients were more predominant in both phases (before: female – 200 and male – 163 vs. after: female – 224 and male – 168). Similar to the control group, most of the patients presented for non-traumatic clinical indications (before: non-trauma – 270 and trauma – 93 vs. after: non-trauma - 309 and trauma - 83).

**Table 2:** Quality of imaging referrals according to RI-RADS (Intervention group)

	RI-RADS grade	*n*/*N* (%)	Presence of clinical findings	Presence of impression	Presence of diagnostic question	Positive	Negative
**Before**							

Cervical spine X-ray	B	2/98 (2.0%)	2/2 (100%)	2/2 (100%)	2/2 (100%)	0/2 (0%)	2/2 (100%)
	C	18/98 (18.4%)	15/18 (83.3%)	9/18 (50.0%)	11/18 (61.1%)	0/18 (0%)	18/18 (100%)
	D	78/98 (79.6%)	74/78 (94.9%)	3/78 (3.8%)	1/78 (1.3%)	0/78 (0%)	78/78 (100%)
Lumbar spine X-ray	B	2/265 (0.8%)	2/2 (100%)	2/2 (100%)	2/2 (100%)	0/2 (0%)	2/2 (100%)
	C	45/265 (17.0%)	43/45 (95.6%)	17/45 (37.8%)	28/45 (62.2%)	5/45 (11.1%)	40/45 (88.9%)
	D	218/265 (82.2%)	207/218 (95.0%)	8/218 (3.7%)	3/218 (1.4%)	26/218 (11.9%)	192/218 (88.1%)

**After**							

Cervical spine X-ray	B	5/110 (4.5%)	5/5 (100%)	5/5 (100%)	5/5 (100%)	0/5 (0%)	5/5 (100%)
	C	23/110 (20.9%)	21/23 (91.3%)	8/23 (34.8%)	14/23 (60.9%)	0/23 (0%)	23/23 (100%)
	D	82/110 (74.6%)	74/82 (90.2%)	4/82 (4.9%)	4/82 (4.9%)	0/82 (0%)	82/82 (100%)
Lumbar spine X-ray	B	5/282 (1.8%)	5/5 (100%)	5/5 (100%)	5/5 (100%)	0/5 (0%)	5/5 (100%)
	C	66/282 (23.4%)	64/66 (97.0%)	25/66 (37.9%)	44/66 (66.7%)	10/66 (15.2%)	56/66 (84.8%)
	D	211/282 (74.8%)	202/211 (95.7%)	6/211 (2.8%)	3/211 (1.4%)	22/211 (10.4%)	189/211 (89.6%)

In the before phase (intervention), there were two cervical and lumbar spine X-ray imaging referrals, respectively, that were classified as *barely adequate* (grade B). There was a high percentage of imaging referrals classified as *deficient* (grade D), which ranged from 79.6% (cervical spine X-ray) to 82.2% (lumbar spine X-ray). No positive findings were identified in the cervical spine X-rays. In contrast, the lumbar spine X-rays graded C and D demonstrated comparable rates of positive findings, with grade C - 11.1% and grade D - 11.9%.

In the after phase, there were 10 imaging referrals (5 cervical and 5 lumbar spine X-rays) that were classified as *barely adequate* (grade B), a modest increase from before. Similar to the “before” phase, imaging referrals of *deficient* (grade D) were predominant (74.6% and 74.8%), and no cervical spine X-rays reported positive findings. Conversely, the rates of positive findings for lumbar spine X-rays were grade C – 15.2% vs. grade D – 10.4%.

### Comparison of RI-RADS grade D proportion after intervention

After intervention (control: no CDSS, routine practice; intervention: CDSS), the proportion of deficient examinations (grade D) did not improve significantly between groups for cervical (CDSS: 74.6% vs. non-CDSS: 77.3%, *p* = 0.753) or lumbar examinations (CDSS: 74.9% vs. non-CDSS: 74.5%, *p* = 1.000) (Table 3).

**Table 3:** Comparison of deficient (grade D) imaging referrals proportion after intervention

Examination/group	Grade	*n*/*N* (%)	Examination/group	Grade	*n*/*N* (%)	*χ*^*2*^	*p*
Cervical/CDSS	Potentially sufficient (A-C)	28/110 (25.45)	Cervical/Non-CDSS	Potentially sufficient (A-C)	25/110 (22.73)	0.099	0.753
Cervical/CDSS	Deficient (D)	82/110 (74.55)	Cervical/Non-CDSS	Deficient (D)	85/110 (77.27)		
Lumbar/CDSS	Potentially sufficient (A-C)	71/282 (25.18)	Lumbar/Non-CDSS	Potentially sufficient (A-C)	62/243 (25.51)	0.000	1.000
Lumbar/CDSS	Deficient (D)	211/282 (74.82)	Lumbar/Non-CDSS	Deficient (D)	181/243 (74.49)		

Dichotomizing RI-RADS Grades into deficient (grade D) versus potentially sufficient (grades A–C).

In the CDSS group, the proportion of deficient imaging referrals (grade D) decreased significantly from 81.5% to 74.7% (*χ*^*2*^ = 4.689, *p* = 0.030), whereas a smaller decrease was observed in the non-CDSS group (80.9%–75.4%, *p* = 0.135). However, logistic regression including study group, time phase, and the group-by-time interaction demonstrated that difference in before-after changes between the two groups was not statistically significant (*p* for interaction = 0.700) (Table 4), suggesting improvement over time without CDSS-specific enhancement.

**Table 4:** Change in proportion of deficient imaging referrals (grade D)

	Before % (*n/N*)	After % (*n/N*)	Change (%)	*p* (within group)	OR *(*95% *CI)*; *p* (group × time interaction)
CDSS	81.54 (296/363)	74.74 (293/392)	−6.80	0.030^a^	1.11 (0.64, 1.94); 0.700
Non-CDSS	80.91 (195/241)	75.35 (266/353)	−5.56	0.135	

*p* (within group) was obtained using Pearson’s chi-square tests comparing before and after intervention proportions within each group.

*p* (interaction) from mixed-effects logistic regression with binomial response: deficient imaging referral (grade D) ∼ study group × time phase + (1|Physician).

a *p* <0.05.

### Mixed-effects logistic regression analysis

An additional mixed-effects logistic regression model, which included study group, study period, gender, trauma, and body region as fixed effects, with physician included as a random intercept to account for clustering by physician, demonstrated no significant differences between the CDSS and non-CDSS groups (odds ratio, OR 0.93, 95% confidence interval, *CI* 0.62–1.39, *p* = 0.726) (Table 5). Body region and gender was not significantly associated with imaging referral quality (lumbar versus cervical spine: OR 0.84, 95% *CI* 0.62–1.14, *p* = 0.266 and male versus female: OR 0.80, 95% *CI* 0.61–1.04, *p* = 0.099). The odds of a deficient imaging referral were significantly lower in September 2025 (OR 0.48, 95% *CI* 0.23–0.99, *p < 0*.*05*) and November 2025 (OR 0.40, 95% *CI* 0.18–0.88, *p < 0*.*05*), compared with May 2025. Trauma-related imaging referrals had significantly higher odds of grade D classification compared with non-trauma-related imaging referrals (OR 1.56, 95% *CI* 1.09–2.23, *p* < 0.05).

**Table 5:** Mixed-effects logistic regression analysis, with physician included as a random intercept to account for clustering

Variable	Adjusted OR	95% *CI*	*p* value
Study group			
Non-CDSS vs. CDSS	0.93	0.62–1.39	0.726
Study period			
Month 2 (June 2025) vs. Month 1 (May 2025)	1.12	0.57–2.22	0.738
Month 3 (July 2025) vs. Month 1 (May 2025)	0.86	0.50–1.47	0.570
Month 4 (August 2025) vs. Month 1 (May 2025)	0.72	0.41–1.26	0.247
Month 5 (September 2025) vs. Month 1 (May 2025)	0.48	0.23–0.99	**0.046**^a^
Month 6 (October 2025) vs. Month 1 (May 2025)	0.59	0.30–1.16	0.122
Month 7 (November 2025) vs. Month 1 (May 2025)	0.40	0.18–0.88	**0.023**^a^
Body region			
Lumbar vs. cervical	0.84	0.62–1.14	0.266
Gender			
Male vs. female	0.80	0.61–1.04	0.099
Trauma			
Trauma vs. non-trauma	1.56	1.09–2.23	**0.014**^a^

Mixed-effects logistic regression model with binomial response: deficient imaging referral (grade D) ∼ study group + study period + body region + gender + trauma + (1|Physician). Physician was included as a random intercept to account for clustering of imaging referrals within physicians. *N* = 1,349 imaging referrals from 40 physicians. OR = odds ratio; CI = confidence interval.

a *p* <0.05.

## Discussion

Our implementation study revealed that approximately three-quarters of ED imaging referrals were classified in the RI-RADS grade D category. The majority of imaging referrals had the description of clinical findings, but information on impression and diagnostic questions was lacking. Additionally, there was no statistical difference in before and after changes between the control group and the intervention group. Both groups showed modest improvements in imaging referrals classified as *deficient* (grade D) before and after, with statistically significant improvements observed in the intervention group. Thus, the results provided insufficient evidence to conclude that the availability of CDSS deployed alongside current workflows was associated with an improvement in imaging referral quality compared to the control group over the same period.

In our implementation study, the availability of non-integrated CDSS was not associated with enhancement in the quality of RI-RADS, potentially due to limitations inherent in its standalone deployment/availability alongside existing clinical workflow. Like many radiology CDSS, the standalone CDSS implemented in this study also required relevant clinical information (Figure 1) to be input before a radiological examination could be requested, allowing for effective clinical decision support. However, a fundamental design weakness in the current standalone CDSS, where entering a clinical diagnosis was not compulsory, may lead to physicians manipulating the system. Physicians could either ignore the clinical diagnosis field or input a period “.” or similar random character to advance to the next step. Indeed, we observed multiple entries without any clinical information input. Such observations highlight a design drawback that warrants attention.

**Figure 1:**
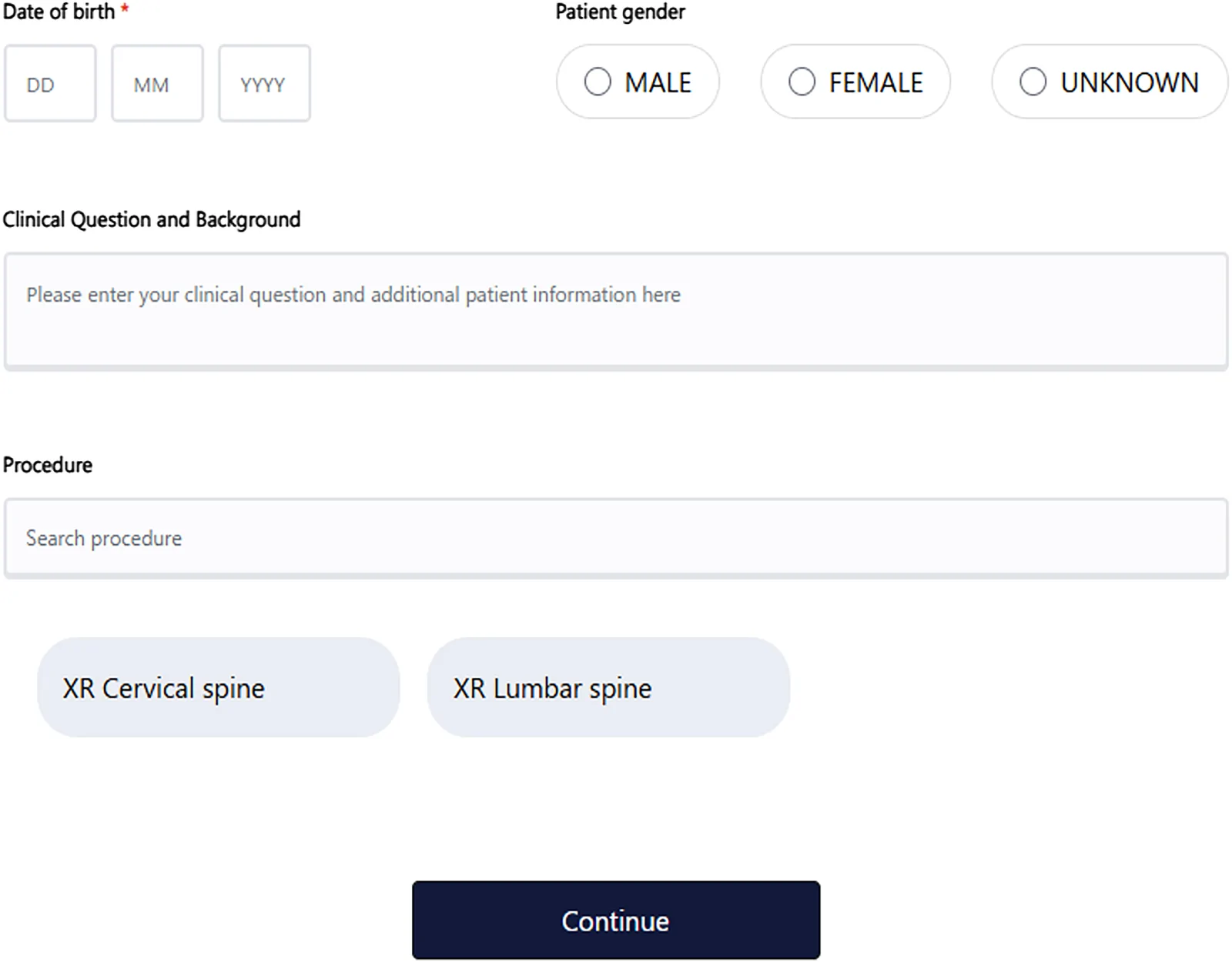
Patient details screen in CDSS for imaging referral Example of the patient details screen within the standalone CDSS for an imaging referral. Physicians are required to enter the patient’s date of birth, select the patient’s gender, enter the clinical question and relevant clinical background, and lastly select the intended imaging procedure (cervical spine X-ray or lumbar spine X-ray) before proceeding with the CDSS workflow.

Another challenge concerning the current CDSS instance revolves around the communication of data within the system. There was no validation of clinical diagnosis with the selection of structured indications. Using cervical spine X-ray as an example, one could input the clinical diagnosis as *“cervicogenic headache”* and select the structured indication *“Trauma (Indicated by NEXUS or CCR clinical criteria)”* resulting in “Indicated” for the imaging referral—a discordant and/or contradiction between the actual clinical indication and selected CDSS indication. As a result, physicians may not feel obligated to complete the clinical diagnosis or improve the quality of information provided.

More critically, the standalone CDSS deployed alongside existing clinical workflow introduces workflow interruption and friction. This led to an implementation approach that failed to sufficiently motivate participating physicians to engage.^12^ This could have contributed to modest uptake of CDSS in our study, where the adoption rate was approximately 47.8%, although a meta-analysis of 55 studies (60 CDSS study arms) by Kouri et al.^13^ revealed that overall weighted CDSS uptake was 34.2% (95% *CI* 23.2 to 47.1%). A CDSS includes not just technical content but also a workflow (Figure 2). Physicians will now need to engage with a separate, standalone CDSS as an extra step in addition to their current imaging referral process via computerized provider order entry (CPOE). This added friction could reduce the time and cognitive bandwidth available for composing detailed free-text clinical information. Without any tangible benefit to the physician (i.e., lack of auto-transportation of clinical information, no feedback flowing back into the ordering process and no enforced alignment between indication and clinical information), any changes in RI-RADS within the intervention group would depend solely on voluntary behavioral change in documentation practices. Similarly, this may have potential implications for imaging appropriateness, physicians’ guidelines, and CDSS recommendation compliance.

**Figure 2:**
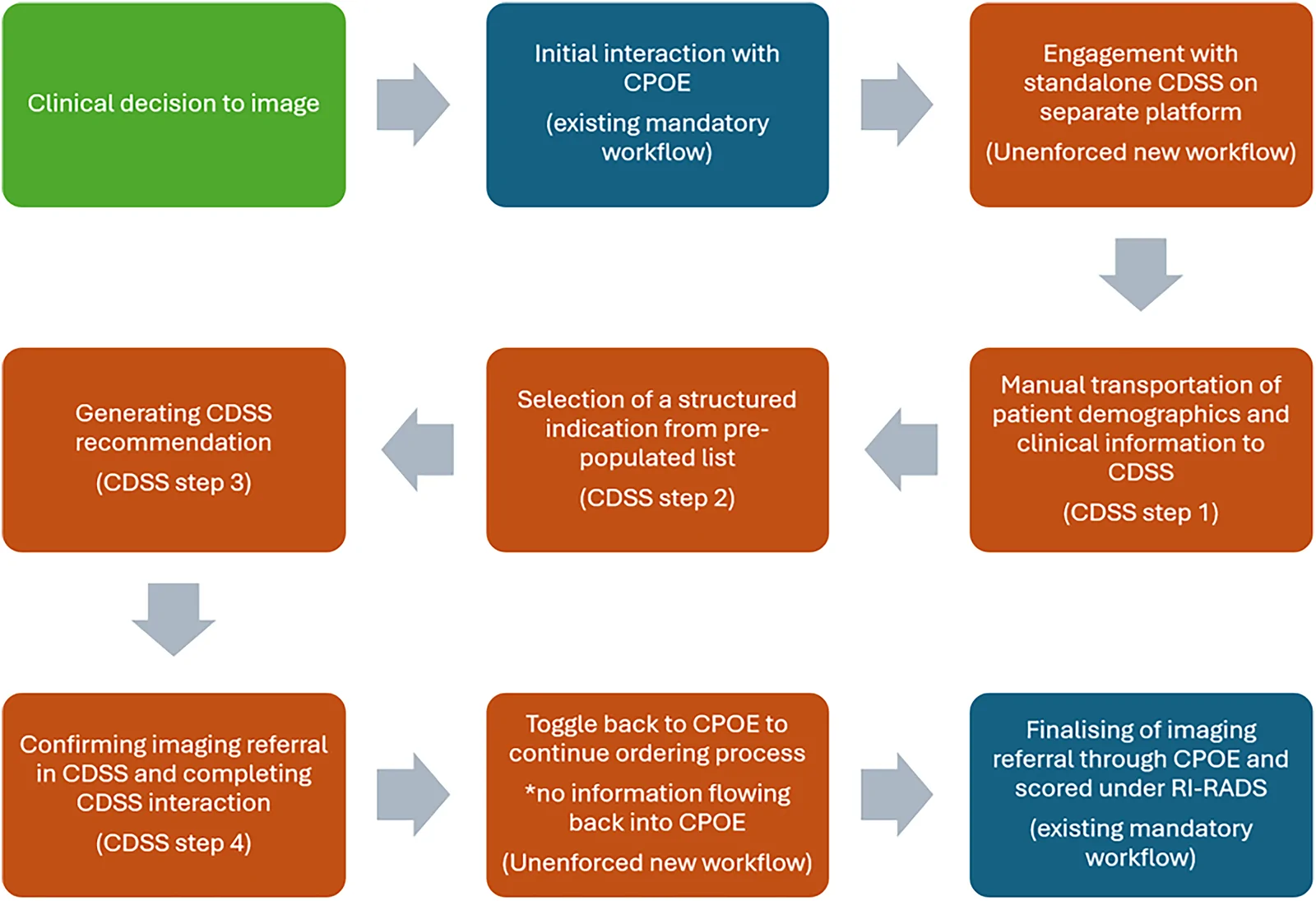
Workflow diagram of standalone CDSS alongside existing workflow Workflow illustrating the standalone CDSS operating as an unenforced new workflow alongside the existing mandatory CPOE workflow. Following the imaging decision, physicians could access the standalone CDSS through a separate platform that was not integrated into CPOE. The CDSS workflow involved manual input of patient demographics and clinical information, selection of a structured clinical indication from a pre-populated list, generation of an imaging recommendation, and confirmation of the imaging referral. Physicians subsequently returned to the mandatory CPOE workflow to complete the request for imaging referral, with no automated transfer of information between the CDSS and CPOE. The completed imaging referral in CPOE was subsequently graded using the RI-RADS.

Our findings corroborate with insights from Australian policymakers, which indicate that workflow disruptions, conflicts with existing workflow dynamics in clinical settings, and insufficient integration of the CDSS knowledge base with patient information limit the impact and success of CDSS implementation.^14^ It also underscores the limitations associated with implementing an external, standalone CDSS in clinical practice, given the workflow disruptions, limited feedback mechanisms, and absence of a significant improvement on imaging referral documentation resulting from our approach.

While integrating the CDSS into electronic health records (EHRs) can resolve some of the aforementioned challenges (i.e., manual transportation of information and absence of information flowing back into CPOE), ultimately, communication and exchange of data should also be built into the CDSS to mitigate gaming of the system. Mandating physicians to enter structured indications at the point of entry would be one approach to facilitate mapping to recommendations from imaging referral guidelines. However, such an approach results in end-user frustration and does not resolve the problem of system gaming, nor contribute to improving the quality of imaging referrals.^15^

Alternatively, artificial intelligence (AI) can be incorporated into the CDSS to enable a free-text AI approach, which facilitates the selection of structured indications for clinical decision support. Such an AI approach has been preferred over a direct search for structured indications, with a study demonstrating that ED physicians were more likely to use it as compared to other settings.^15^ Nevertheless, this approach still does not address the issue of low-quality imaging referrals.

In the era of AI, named entity recognition (NER), which is a form of natural language processing, can help identify and categorize key information. With an integrated CDSS, NER can potentially extract input from physicians in free text (such as diseases, signs and symptoms) within EHR at scale, reshape it into structured clinical information, and use it for clinical decision support without requiring physicians to manually input clinical diagnoses.^16^ This addresses the various concerns about the quality of imaging referrals and system gaming while reducing physicians’ workloads and time spent on CDSS engagement. Moreover, with the integration of timely and comprehensive feedback on an individual’s performance, individuals can improve their practice, thus contributing to advancements in patient care and the downstream value of radiology.^17^

However, a culture of feedback is paramount for any form of feedback mechanism to be effective.^17^ Leaders in the institution should embrace and embed a culture of feedback, and in our context, within the ED. Moreover, feedback needs to be acted upon and monitored as part of the improvement follow-up. A systematic review has shown that the most consistent effective intervention to improve the quality of imaging referrals was to employ penalties.^18^ Indeed, to promote quality within the department, it may be necessary to introduce a system of carrot and stick, but it is important to implement these measures appropriately to avoid any negative consequences.^19^

Our findings were consistent with available literature and raised concerns about the potential implications of insufficient pertinent information in ED imaging referrals. Studies from the Netherlands reported inadequate imaging referral information in >75% of cases,^20^ while >50% of the imaging referrals from a single center in Italy were assessed to be deficient.^21^ Additionally, a recent study evaluating emergency CT (computed tomography) referrals yielded similar outcomes where most imaging referrals were inadequate. Notably, imaging referrals with lower quality according to RI-RADS were associated with worse clinical reasoning quality.^22^ This result reflects the implications of low-quality imaging referrals for patient safety and effective patient care.

Poorly justified imaging referrals may result in an inappropriate examination, which could completely hide a significant abnormality and negatively affect patient outcomes.^23^ For instance, radiographers use the referral information for justifying and optimizing imaging examinations; the absence of this crucial information can impact patient safety and the provision of services.^24^ Likewise, insufficient clinical information affects the productivity of clinical practice in radiology departments, as examining radiographers and radiologists may have to spend more time gathering and clarifying referral details to justify imaging, such as through a phone call or a visit to the consultation room.^25^

Consequently, in actual practice and during the study itself, the quality of imaging referrals does not necessarily affect the “acceptance” of imaging referrals by radiographers. Radiographers in such instances will routinely contact the referring physicians for more information to clarify and justify the examination. This form of informal learning among the ED physicians could also be a plausible reason for improvements in imaging referral quality without CDSS intervention.

Furthermore, insufficient clinical information provided by physicians can increase the rate of interpretation error.^26^ This is concerning in our context, where impression and diagnostic questions are substantially lacking in the majority of the imaging referrals, though they are in line with findings from the literature.^21^ Additionally, positive radiological findings (findings that impact patient management) were extensively reported in examinations classified as *deficient* or *considerably limited* (e.g., lower back pain; fall). With such imaging referral patterns, we may be subjecting patients to fallacious interpretation.^27^ Moreover, radiologists may struggle to furnish adequate context in radiological reports due to a lack of relevant background information, hence impacting physicians’ satisfaction with the completeness or quality of these reports.^3^^,^^28^ Although beyond the scope of this study, it would be interesting to explore the proportion of imaging referrals that subsequently ended with follow-up imaging due to inadequate clinical information in the imaging referrals.

Conversely, imaging referrals that lacked quality may also be clinically inappropriate. Parillo et al.^29^ evaluated head CT referral quality and appropriateness in an Italian ED and observed that most ED head CT referrals lacked quality and were often clinically inappropriate. This observation warrants attention, as it suggests that a significant number of imaging referrals in our study may be clinically inappropriate. However, due to resource constraints, our study did not specifically examine imaging appropriateness rates and guideline concordance.

This study adopted a pragmatic controlled before-after approach in evaluating CDSS availability alongside existing workflow on the quality of imaging referrals. We minimized the risk of bias by ensuring that unidentified differences between the control and intervention groups that may affect changes in the outcome measurement were addressed through recruitment strategies. Additionally, there was no other local intervention that may have influenced the results. We also reported their baseline outcome measurements for all to assess for similarity and comparability. Simultaneously, we used RI-RADS, which is a standardized method for evaluation of the quality of information in an imaging referral, facilitating comparison with findings from other studies adopting RI-RADS. Nonetheless, the Hawthorne effect may have influenced physicians’ behavior, as they were informed that imaging referrals would be audited and monitored during the study (CDSS).^30^ However, because the outcome measures did not emphasize the quality of imaging referrals, we postulate that the influence of the Hawthorne effect would be modest.

In our actual implementation, we recruited a total of 1,349 patients clustered within 40 unique physicians. The average cluster size (*m*) was approximately 33.7 imaging referrals per physician (1,349/40). In the absence of a directly applicable intraclass correlation coefficient (ICC) estimate for imaging referrals quality clustered at the physician level, an ICC of 0.03 was assumed for post hoc sensitivity analysis, consistent with the ICC reported in a previous randomized trial evaluating radiological CDSS-related imaging appropriateness outcomes.^31^ Moreover, the estimated ICC of 0.03 is a value commonly used in cluster-randomized trials.^32^ Using an ICC of 0.03, the calculated design effect (DE = 1 + [*m* – 1] ∗ ICC = 1.982) would correspond to a cluster-adjusted sample size of approximately 468 ∗ 1.982 = 928 imaging referrals. Additional sensitivity analyses were performed using ICC values of 0.01 and 0.05 to assess the potential impact of physician-level clustering. Assuming ICC = 0.01, the cluster-adjusted sample size was approximately 622 imaging referrals, while ICC = 0.05 was approximately 1,234 imaging referrals. The retrospective nature of these calculations raises uncertainty regarding the adequacy of statistical power when accounting for physician-level clustering.

In the future, cluster-randomized, stepped-wedge trials should be conducted with CDSS integrated into EHR and clinical workflow while incorporating hard stops aligned to RI-RADS, AI-assisted auto-population, and feedback interventions. Evaluations of downstream outcomes, such as additional imaging, ED length of stay, time-to-diagnosis, and revisit rates, should be performed to provide further insights. It would also be interesting to evaluate mixed-effects modeling with patient and physician covariates, including advanced imaging, particularly when our local institution’s workflow involves seeking a radiologist’s justification or approval for advanced imaging. The findings may have implications for future workflow, such as automated approval of advanced imaging examinations if justified by CDSS.

In conclusion, our implementation study demonstrated an absence of a statistically significant between-group effect. However, given the non-randomized design, incomplete concurrence of the comparison periods, and absence of objective CDSS utilization data, temporal confounding cannot be excluded, precluding firm conclusions about the CDSS’s effectiveness. Consequently, availability of CDSS was not associated with benefit beyond secular improvement and reinforced the realities of standalone CDSS deployed alongside existing workflows. The CDSS standalone nature coupled with limitations of its design and the use of structured indications may have limited its potential in enhancing the quality of imaging referrals. With only modest self-reported use of CDSS, future CDSS should be integrated seamlessly with health technologies such as EHR and CPOE, into clinical workflow, and design and implementation should take these factors into account to enhance the quality of imaging referrals and promote value-added radiology in healthcare.

### Limitations of the study

This study has several limitations. First, since our study included only one institution’s ED, the results may be confounded by the specific site, which limits generalizability. However, we had taken steps to ensure that the observed differences were attributable to the intervention rather than other variables. Additionally, although the study was conducted in a real-world setting, the focus on a specific demographic, junior ED physicians, means that our findings may not be applicable to all contexts. Similarly, our findings from X-ray imaging may not be applicable to advanced imaging techniques, which are often more detailed due to the nature of the imaging referral, as well as to integrated CDSS models and healthcare systems outside the study institution.

Second, the data were collected from junior ED physicians and may not represent the behaviors of more experienced senior ED physicians or ED specialists. Nonetheless, there may be potential selection bias in physician assignment though we allocated them based on assigned roster. Additionally, as we did not collect characteristics of participants, physician experience was not considered in the analysis, and this may result in a potential influence of baseline group differences on outcomes. In a similar vein, due to zero cell counts in certain subgroups (grades A and B = 0 in the non-CDSS group), methods such as ordinal analysis would lack statistical comparability across groups, necessitating the consolidation of grades A–C. Consequently, our specific approach to the dichotomization of grades D versus A–C may influence the outcome measure, which represents another limitation of this study.

Next, the adoption rate of our study reflects that our implementation approach may not equally motivate all physicians to participate, resulting in data noise.^12^ Since we did not track the actual CDSS utilization rate at the physician level, our findings likely reflect the outcome measure associated with the availability of CDSS rather than the actual CDSS exposure/impact. Additionally, our initial *a priori* sample size calculation did not account for physician-level clustering which represents an additional limitation of this study.

Finally, our study may be subjected to secular trends and non-concurrent periods. Simultaneously, as patient identifiers were unavailable, adjustment for patient-level case-mix and clustering could not be performed, potentially resulting in an underestimation of variance and reduced ability to detect modest between-group differences. Additionally, although referrals were independently assessed by two auditors with almost perfect inter-rater agreement, the possibility of bias arising from incomplete blinding cannot be excluded, as one assessor was aware of the study group and time.

## Resource availability

### Lead contact

• Requests for further information and resources should be directed to and will be fulfilled by the lead contact, Yi Xiang Tay (yi.tay@ucdconnect.ie).

### Materials availability

• This study did not generate new unique reagents.

### Data and code availability

• The RI-RADS grading data and associated imaging referral datasets generated and/or analyzed during the current study are not publicly available because they contain sensitive patient clinical information and are subject to institutional data-governance and confidentiality requirements. A de-identified dataset containing the variables necessary to reproduce the principal analyses may be made available from the lead contact upon reasonable request, subject to institutional approval and applicable data-sharing requirements, including de-identification through an independent trusted third party where required. Statistical analysis scripts may also be made available from the lead contact upon reasonable request.
• This study does not report original code.
• Any additional information required to reanalyze the data reported in this study may be made available from the lead contact upon reasonable request.

## Acknowledgments

This work was supported by the SingHealth Talent Development Fund and 10.13039/501100001469Singapore General Hospital Scholarship.

## Author contributions

Conceptualization, investigation, and methodology, Y.X.T.; formal analysis, Y.X.T., W.H., B.W., and J.P.M.; writing – original draft, Y.X.T. and J.P.M.; writing – review and editing, W.H., S.J.F., R.K., M.E.H.O., J.C.P.W., I.S.Y.C., B.W., P.D., R.C.C., and L.P.C.; supervision, S.J.F., R.K., M.E.H.O., and J.P.M.

## Declaration of interests

While R.K. serves as Chief Medical Officer at xWave Technologies, it is important to note that the results of the study were not contingent on the industry partner’s approval or censorship. This independence extended to data analysis, interpretation, and the reporting of results. R.K. recused himself from any decision-making, with Y.X.T. and J.P.M. designated as the primary contacts for communication with the industry partner. R.K. was only involved in supervising Y.X.T. as one of the PhD co-supervisors and in the manuscript review and editing. R.K. did not make any decision that involved xWave Technologies, with decision-making involving only Y.X.T. and J.P.M. (the PhD primary supervisor and senior author). xWave Technologies appointed a project manager for the study, who liaised directly with Y.X.T. to facilitate modifications to the user interface. It is noteworthy that xWave Technologies acted solely as a service provider in this study, specifically to assist in making the standalone CDSS available commercially.

## STAR★Methods

### Key resources table

**Table:** 

REAGENT or RESOURCE	SOURCE	IDENTIFIER
**Software and algorithms**		

R version 4.5.2	R Foundation	https://www.r-project.org/

**Other**		

Reason for exam Imaging Reporting and Data System (RI-RADS): A grading system to standardize radiology requisitions	Abedi et al.^7^	https://doi.org/10.1016/j.ejrad.2019.108661
Optimising design of clinical decision support systems and implementation strategies to improve radiological imaging appropriateness	Tay et al.^9^	https://doi.org/10.1016/j.ijmedinf.2025.105966
Study protocol for CDSS implementation	Tay et al.^33^	https://doi.org/10.1371/journal.pone.0336801
Assessing context and readiness of emergency medicine physicians to promote evidence-based imaging referral guidelines	Tay et al.^34^	https://doi.org/10.1093/tbm/ibaf035

### Experimental model and study participant details

#### Participant details

A purposive sample of non-specialist ED doctors (medical officers, locum medical officers and emergency medicine residents) was recruited by the ED trauma lead and a representative from the ED Trauma committee as they accounted for the majority of X-ray imaging referrals in the ED.

#### Patient cohort details

Imaging referrals quality was assessed for 1,349 patients presenting to the ED with valid cervical or lumbar spine X-rays. In the control group, 241 and 353 patients were included in the before and after phases, respectively. Their mean age was 61.7 ± 17.4 (before) and 58.4 ± 18.8 (after). In the intervention group, 363 and 392 patients were included in the before and after phases, respectively. For this group, their mean age was 58.6 ± 19.6 (before) and 58.8 ± 19.7 (after).

#### Institutional permission and oversight information

Ethics and informed consent waivers were granted by the SingHealth Centralised Institutional Review Board (No. 2020/2941), as the study was classified a quality assurance audit. Cybersecurity approval was obtained from the institution’s information technology (IT) team for the implementation of the CDSS in the ED (SGH-250034).

### Method details

#### Study protocol

The associated study protocol for the implementation of CDSS was published in 2025, with changes made to account for a transition period before the evaluation of impact.^33^

#### Study design

This implementation study was conducted using a pragmatic before-and-after design. This study did not use randomisation or blinding due to practical implementation challenges. To increase adoption and effectiveness, elements from an implementation science process model, including the engagement of change agents (authoritative leaderships), context analysis, and practice change approach, were integrated into the implementation. More details on the context analysis and strategies can be found in our publications.^9^^,^^34^

The ED physicians were allocated to the control group (non-CDSS) or the intervention group (CDSS) based on their assigned rosters (Figure 3). Both groups had a “before” evaluation period of two months (routine practice; no engagement with CDSS), which was followed by another two months of evaluation as part of the “after” phase (control: routine practice; no engagement with CDSS and intervention: engagement with CDSS) (Figure 3). Both groups’ evaluations took place between May and November 2025, with the control group study duration being non-CDSS (before): 01 May to 30 June and non-CDSS (after): 01 July to 31 August and the intervention group non-CDSS (before): 01 July to 31 August and CDSS (after): 15 September to 14 November.

**Figure 3:**
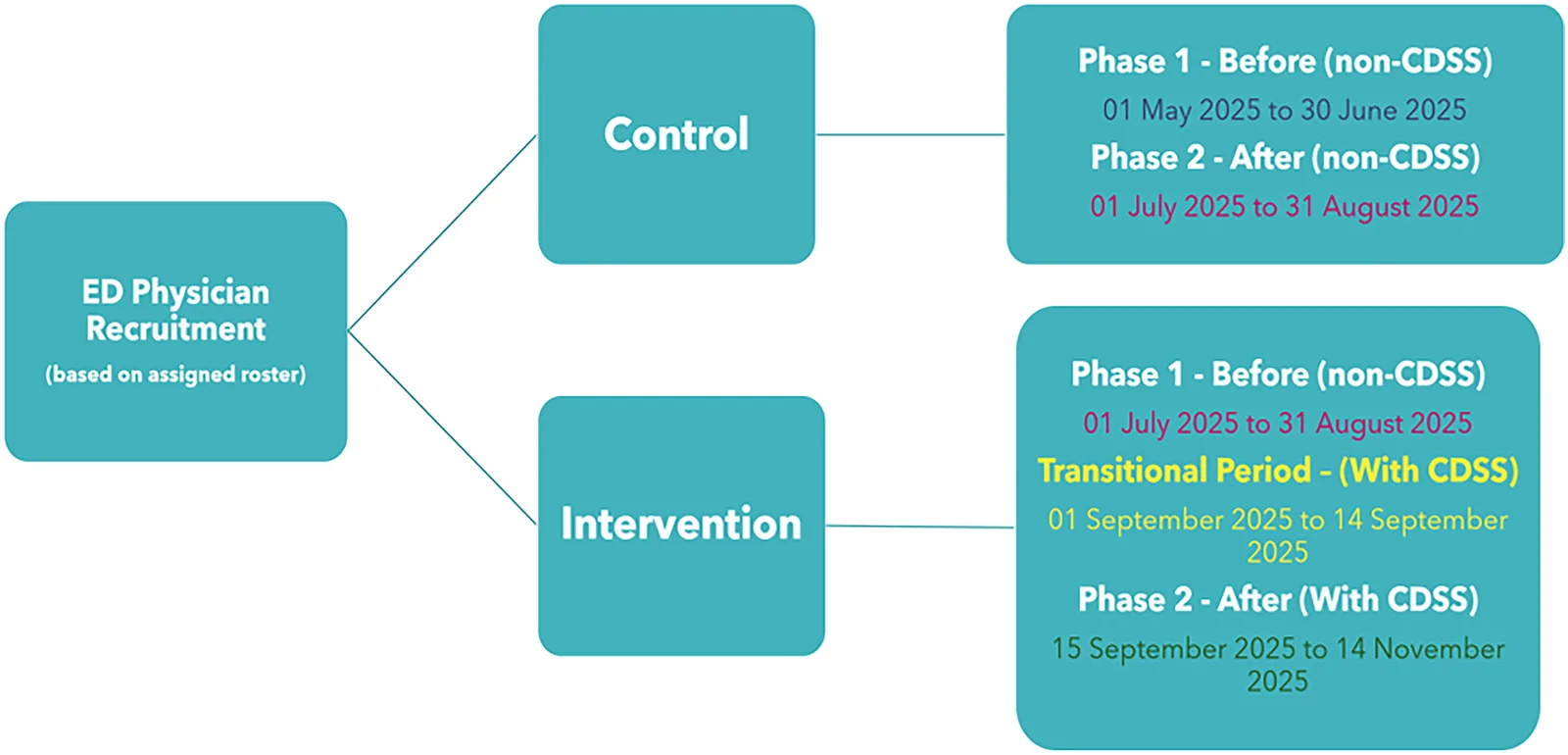
Timeline of control and intervention group before-after Timeline illustrating the study periods for the control (non-CDSS) and intervention (CDSS) groups, comprising a 2-month before period and a 2-month after period for both groups. A 2-week transition period was included for the intervention group between the before and after periods to allow physicians to acclimatize to the standalone CDSS and its use alongside the existing clinical workflow, while enabling the study team to observe CDSS adoption and compliance with the prescribed work procedures. During the after period, the intervention group had access to the CDSS, while the control group continued with the existing imaging referral workflow without CDSS availability.

The period spanning from 01 September to 14 September was designated as a period of transition for ED physicians to acclimatize to the CDSS, enabling the study team to observe their adoption rates and compliance with prescribed work procedures. Prompt intervention via reminders in physicians’ chat groups and from senior physicians was initiated upon the identification of shortcomings during the initial monitoring phase utilising the CDSS dashboard.

#### Study setting

The study was conducted in the ED of Singapore General Hospital. A standalone CDSS (xWave Technologies, Ireland, Dublin) was deployed with institutional medical board-approved imaging referral guidelines embedded within. Modifications were made to the user interface to accommodate feedback from end-users as highlighted in previous local studies.^9^^,^^34^ In view of an impending information technology infrastructure transition within the hospital, integration of CDSS with computerised provider order entry (CPOE) was not possible, and therefore the intervention took place as a standalone model which required logging into a separate web-based CDSS. Physicians involved in the intervention group were provided dedicated self-directed training materials on the user workflow, orientation of CDSS inputs and the recommendation screen, including an option to attend an onboarding orientation conducted virtually. They were also provided with evidence regarding the positive impact of imaging referral guidelines on the rates of significant positive findings among local patients.^35^

#### Study approach

Structured clinical indications (Figure 4) for selection within the CDSS were populated for cervical and lumbar spine X-rays, in relation to locally approved medical board imaging referral guidelines, which were reviewed by radiologists and senior ED physicians. The process also took reference from previous local studies that explored the appropriateness of these imaging referrals to ensure adequacy of the finalised clinical indications.^36^^,^^37^ Despite the CDSS’s use of structured indications, physicians were reminded that it was also mandatory to enter appropriate and complete clinical questions and background information in the patient details screen (Figure 1) as an initial step before proceeding to the structured clinical indications screen (e.g., copying input data as documented in CPOE).

**Figure 4:**
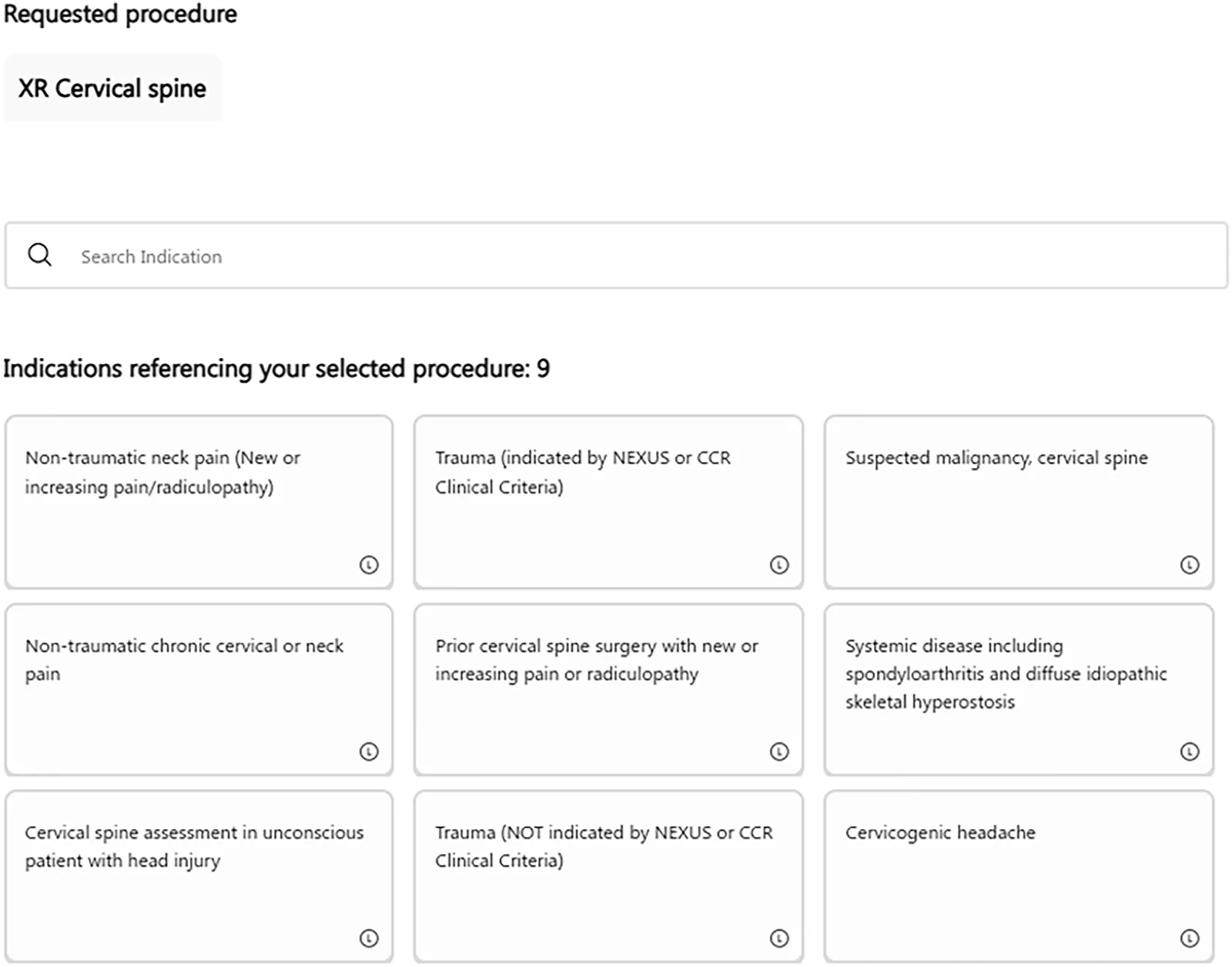
Populated cervical spine X-ray structured clinical indications on CDSS user-interface Example of the standalone CDSS user-interface displaying populated structured clinical indications for a cervical spine X-ray referral. Physicians select the most appropriate clinical indication based on the patient’s clinical presentation, following which the corresponding imaging recommendations are displayed by the CDSS.

The study included consecutive patients referred to the ED’s radiology department for cervical and lumbar spine radiography by the cohort of recruited ED physicians. Patients who received imaging referrals from general practitioners were omitted. Conversely, repeat referrals from same patient were included. For all imaging referrals, the reasons for referral (clinical information), the ordered examination and radiological reports were acquired. The quality of each imaging referral was assessed based on RI-RADS (Table 6). Every referral was assessed by two auditors (Y.X. and J.M.) based on the final referral text in the CPOE system. There was almost perfect agreement between the two auditors’ ratings, *κ* = 0.959, *p* < 0.001. Any disagreements were resolved through discussion and consensus between the two auditors. No gradings required arbitration via a third neutral auditor. The consensus grade was used for the final analysis. Of note, as Y.X. collated all data for the study, he was not blinded to study group, time, physician identity and radiological findings; however, J.M. was blinded to all variables.

**Table 6:** RI-RADS grades, classification, and key categories of information

Grade	Classification	Information included in imaging referral	Key categories of information	Examples of anonymized referral text
A	Adequate	All key categories of information	1. Clinical findings 2. Impression 3. Diagnostic question	ND
B	Barely adequate	All key categories of information, some clinical findings missing	1. Clinical findings 2. Impression 3. Diagnostic question	• Worsening cervical radiculopathy pain after RTA. Bilateral upper limb weakness. TRO fracture. • Left lower back pain, likely MSK. TRO fracture – nil lumbar midline or paraspinal tenderness
C	Considerably limited	Two categories of information	1. Clinical findings 2. Impression 3. Diagnostic question	• Bilateral upper limb weakness. TRO radiculopathy • Fall with lower back pain. TRO fracture
D	Deficient	One or no category of information	1. Clinical findings 2. Impression 3. Diagnostic question	• Neck pain • Low back pain

Note: Adapted from: Abedi A et al. (2019) Reason for exam Imaging Reporting and Data System (RI-RADS): A grading system to standardize radiology requisitions. Eur J Radiol.120:108661.^7^

We classified positive radiological findings as fractures, metastases, and pre-vertebral soft tissue swelling; everything else was negative (i.e., findings that will change patient management in the ED setting).

#### Sample size

Based on previous studies, we estimated that approximately 60% of imaging referrals would be deficient in quality. The unadjusted *a priori* sample size calculation was 468 imaging referrals, to achieve 90% power at a two-sided alpha level of 0.05, assuming a standardised difference between proportions (Cohen’s *h* = 0.30). Since physician-level clustering was not incorporated into the sample size calculation, the expected cluster size, number of clusters, and Intraclass Correlation Coefficient (ICC) were not predetermined.

### Quantification and statistical analysis

For statistical analysis, categorical data were presented as frequencies and percentages, describing the distribution of RI-RADS grades by study group, X-ray examinations and positive/negative rates, both before and after the intervention. Unlike Liu et al.^22^ that classified Grades A-B as adequate and Grades C-D as inadequate, due to sparse data in some RI-RADS classifications, we dichotomised RI-RADS grades as “deficient” (Grade D) versus “potentially sufficient” (Grades A-C). This was supported by data from a recent study,^21^ where Grade D accounted for approximately 53% of the total imaging referrals analyzed, with only 1% for Grade A and 7% for Grade B. Additionally, Grade C imaging referrals were considered limited rather than deficient or incomplete.^38^ Consequently, Grade D was deemed to have significant deficiencies in terms of information completeness by a previous study.^21^

The Chi-squared (*χ*^*2*^) test was used to compare the grade distribution after intervention between the CDSS and non-CDSS groups for each examination. To evaluate the effect of the intervention on the distribution of RI-RADS grades, we analyzed within-group changes from pre-to post-intervention. Between-group differences in these changes were assessed using a logistic regression model that included study group, time phase and time interaction term, with physician included as a random intercept to account for physician-level clustering. Additional mixed-effects logistic regression analysis incorporating study group, study period, body region, gender and trauma, with physician included as a random intercept was also performed. All analyses were performed using R 4.5.2. A two-sided *p*-value <0.05 was considered statistically significant. Our primary hypothesis was that CDSS reduces the proportion of Grade D imaging referrals versus control in the after period. Secondarily, within-group reduction in Grade D from before to after is greater in the CDSS group, with improvements differing by body region.
